# Generation of potent neutralizing human monoclonal antibodies against cytomegalovirus infection from immune B cells

**DOI:** 10.1186/1472-6750-8-85

**Published:** 2008-11-12

**Authors:** Ada Funaro, Giorgio Gribaudo, Anna Luganini, Erika Ortolan, Nicola Lo Buono, Elisa Vicenzi, Luca Cassetta, Santo Landolfo, Richard Buick, Luca Falciola, Marianne Murphy, Gianni Garotta, Fabio Malavasi

**Affiliations:** 1 Department of Genetics, Biology and Biochemistry, University of Torino Medical School, Via Santena 19, 10126 Torino, Italy; 2 Research Center on Experimental Medicine (CeRMS), University of Torino Medical School, Via Santena 19, 10126 Torino, Italy; 3 Department of Public Health and Microbiology, University of Torino, Via Santena 9, 10126 Torino, Italy; 4 Viral Pathogens and Biosafety Unit, DIBIT-San Raffaele Scientific Institute, Via Olgettina 58, 20123 Milano, Italy; 5 Fusion Antibodies Ltd, Pembroke Loop Road, Dunmurry, Belfast, BT17 0QL, Northern Ireland; 6 RiboVax Biotechnologies, 12 Avenue des Morgines, 1213 Petit-Lancy, Geneva, Switzerland

## Abstract

**Background:**

Human monoclonal antibodies (mAbs) generated as a result of the immune response are likely to be the most effective therapeutic antibodies, particularly in the case of infectious diseases against which the immune response is protective.

Human cytomegalovirus (HCMV) is an ubiquitous opportunistic virus that is the most serious pathogenic agent in transplant patients. The available therapeutic armamentarium (e.g. HCMV hyperimmune globulins or antivirals) is associated with severe side effects and the emergence of drug-resistant strains; therefore, neutralizing human mAb may be a decisive alternative in the prevention of primary and re-activated HCMV infections in these patients.

**Results:**

The purpose of this study was to generate neutralizing mAb against HCMV from the immunological repertoire of immune donors. To this aim, we designed an efficient technology relying on two discrete and sequential steps: first, human B-lymphocytes are stimulated with TLR9-agonists and IL-2; second, after both additives are removed, the cells are infected with EBV. Using this strategy we obtained 29 clones secreting IgG neutralizing the HCMV infectivity; four among these were further characterized. All of the mAbs neutralize the infection in different combinations of HCMV strains and target cells, with a potency ~20 fold higher than that of the HCMV hyperimmune globulins, currently used in transplant recipients. Recombinant human monoclonal IgG1 suitable as a prophylactic or therapeutic tool in clinical applications has been generated.

**Conclusion:**

The technology described has proven to be more reproducible, efficient and rapid than previously reported techniques, and can be adopted at low overall costs by any cell biology laboratory for the development of fully human mAbs for immunotherapeutic uses.

## Background

Antibodies constitute the most rapidly growing class of human therapeutics and the second largest class of drugs after vaccines [1]. Most of the growing number of antibodies entering the clinical trials are human [2] and are derived from phage-display technology [3] or from transgenic mice that express human immmunoglobulin genes [4]. However, the best mAbs for clinical applications derive from natural human antibodies generated as a result of the *in vivo *immune response because they i) are products of the human and not animal repertoire, and ii) are of human origin, thus minimizing the risks of reactivity against self-antigens. Lastly, iii) passive immunotherapy with human IgG can confer immediate protection without the side effects linked to the use of chimeric or humanized mAbs containing animal-derived amino acid sequences. Furthermore, considerable evidence indicates that antibodies represent a new, although historically validated, approach to the development of therapies against bacterial and viral pathogens that causes disease in individuals with impaired immune response and/or for which there are few or no available drugs [5-8].

EBV has long been used to immortalize human B-lymphocytes *in vitro *and to isolate human mAb [9-11], but the method was not routinely embraced due to its poor efficiency and because the resulting transformed cells are frequently unstable and fail to grow or secrete IgG. Agonists of Toll receptor 9 (TLR9), *e.g*., CpG ODN 2006 (synthetic oligodeoxynucleotides that contain immunostimulatory deoxycytidyl-deoxyguanosine motifs), have been reported to improve EBV infectivity and cloning efficiency [12], however, there is no clear consensus as to the best conditions for their use and which subset of B-lymphocytes should be used. We applied the EBV immortalization procedure in the presence of different amounts of CpG with or without IL-2 to 3 healthy donors selected on the basis of the high titer of CMV-specific IgG in the blood and we obtained an immortalization efficiency not comparable to that recently reported [12]. Therefore we re-evaluated all the steps crucial to cell immortalization, and established an optimized procedure of isolating human mAbs from the repertoire of immune donors. The resulting method is highly reproducible, effective and rapid. Further, it was validated by the successful isolation of clones of human B-lymphocytes that secrete high levels of IgG that bind and neutralize the human cytomegalovirus (HCMV). HCMV is a ubiquitous opportunistic virus that infects 50–90% of adult human populations and persists for the life of the human host after primary infection. HCMV infection of an immunocompetent individual generally results in subclinical disease; by contrast, HCMV infection of immunocompromised individuals may cause lethal infections [13]. Indeed, HCMV remains the single most important pathogen in hematopoietic stem cell transplantation [14] as well as, in organ transplantation [15] so that effective neutralizing mAbs would make a major contribution to eliminate HCMV in these patients.

## Results and discussion

The isolation of fully human mAb from those generated during the natural human immune response is likely to identify the most effective therapeutic antibodies [6,16]. Many approaches have been used to generate human mAb [17], however, an efficient and reproducible process to isolate human mAb with desired specificity from the natural repertoire is not available.

**Figure 1:**
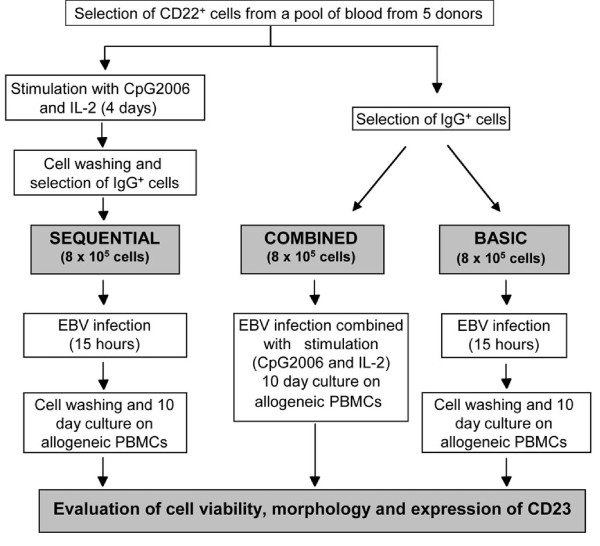
**Outline of the procedure for comparing EBV transformation methods**. Overview of the procedure for comparing the populations of EBV-transformed cells according to the SEQUENTIAL, COMBINED, and BASIC methods.

**Figure 2:**
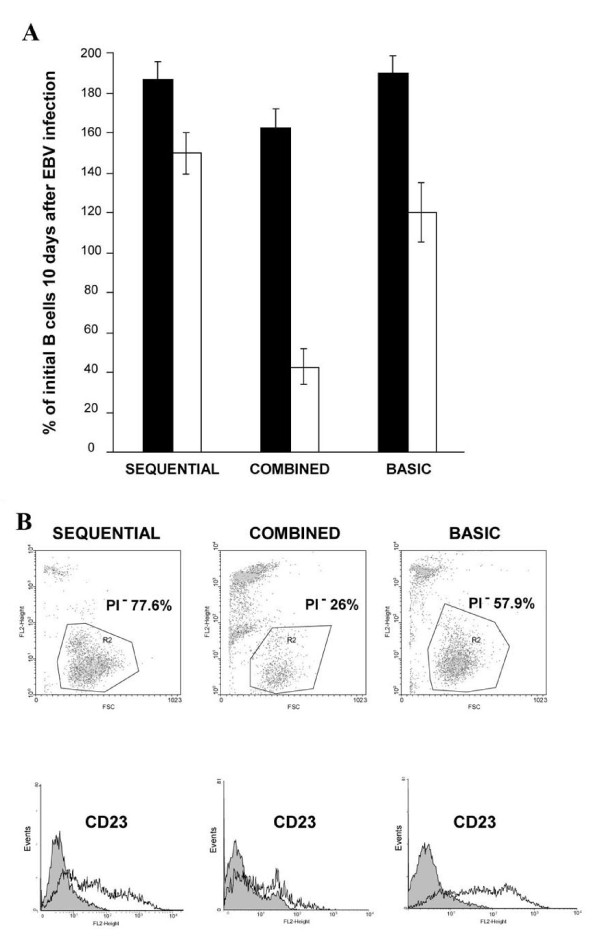
**Quantitative and qualitative comparison of different EBV transformation methods**. A) The B-lymphocyte subsets were prepared using the SEQUENTIAL, COMBINED or BASIC methods, as outlined in Fig. 1. Ten days after exposure to EBV, samples of each population were compared for the total cell number (black bars), and for the number of viable cells (white bars) measured microscopically by trypan blue dye exclusion. Data are expressed as % of initial B cells exposed to EBV and represent the means ± s.d. of 5 cell counts for each condition. Results are representative of two independent experiments. *P *< 0.001: SEQUENTIAL *vs*. COMBINED; BASIC *vs*. COMBINED (white bars); *P *< 0.05: SEQUENTIAL *vs*. BASIC (white bars) and SEQUENTIAL *vs*. COMBINED; and BASIC *vs*. COMBINED (black bars). B) Ten days after infection with EBV, the cells prepared by the SEQUENTIAL, COMBINED, or BASIC method were analyzed to identify viable lymphoblasts by propodium iodide (PI) exclusion (top panels) and CD23 expression (bottom panels) by flow cytometry. For each population of cells, the viable lymphoblasts (gated in regions R2, top panels) were defined as those with relatively high forward scatter (horizontal axes) and negative for PI fluorescence (vertical axes). R2 gated cells were then analyzed for CD23 expression. Fluorescence was analyzed with a FACSCalibur flow cytometer and CellQuest software (Becton Dickinson). Background fluorescence was determined with a FITC-labeled, isotype-matched negative control mAb. 10,000 events were measured for each condition.

Agonists of TLR9 have been reported to improve EBV infectivity and cloning efficiency [12]. However, it is known that TLR9 agonists, such as CpG ODN 2006, protect against a wide range of viral pathogens [18-20]. With this in mind, we reasoned that the presence of CpG during the EBV infection might exert negative effects and we decided to separate activation and EBV infection in two sequential phases. We developed a technology for raising human mAbs for clinical applications based on two discrete steps (SEQUENTIAL, Figure 1): first, purified human B-lymphocytes are treated with CpG2006 and IL-2. Next, after both additives are removed, the activated cells are infected with EBV in a distinct step. The new process was compared to a method using CpG2006 and IL-2 in the presence of EBV [12] (COMBINED) and a method using EBV alone (BASIC) (Figure 1). Different experimental settings were compared in pools of cells derived from 5 healthy donors, to minimize donor variability. The results clearly demonstrated that activation of CD22^+ ^B-lymphocytes before EBV exposure leads to improved immortalization, as highlighted by the viability of cells following EBV transformation which is significantly higher than that obtained with both combined and basic conditions (Figure 2A). The short-term viability of B lymphocytes immortalized following the basic procedure is higher than those of B lymphocytes treated with the combined procedure. This is attributable to the polyclonal activation elicited by EBV itself which is not paralleled by an efficient immortalization. Indeed, it is well documented that exposure of human B-lymphocytes to EBV results in cell death of a proportion of cells and that B-lymphocytes are activated but not immediately immortalized following exposure to EBV [21-23].

Removal of the activating agents before immortalization significantly improves growth and viability of CD22^+ ^B-lymphocytes allowing for a higher proportion of cells to be immortalized. Indeed, when stimulation and EBV infection are performed separately rather than simultaneously, or with no stimulation at all, the cells exhibit increased proliferation potential and improved viability. In contrast, the combined presence of activating agents and EBV has adverse effects on both cell viability and EBV infectivity (Figure 2A, B). The negative effects of the COMBINED method on viability are surprising, given that CpG2006 induces robust polyclonal activation and proliferation of B-lymphocytes [24,25] and that these effects are significantly enhanced by IL-2 [26]. It has been reported that TLR-9 triggering resulted in higher transformation rates of B cells infected with EBV [12] but a comparison between EBV transformation in the presence of CpG2006 and EBV and after sequential exposure of B cells to CpG2006 and separately to EBV, was not investigated. The results obtained using different experimental setting on the same cell population clearly demonstrated that CpG2006 and IL-2 used in combination with EBV exert negative effects on cell transformation. It is conceivable that the detrimental effects are related to the anti-proliferative activity of anti-viral cytokines induced by CpG2006 [27,28]. Indeed, the significant levels of TLR9 expressed by resting B-lymphocytes are further up-regulated by CpG2006 itself. Increased TLR9 expression correlates with increased responsiveness to the ligand, indicating a positive feedback loop in which CpG2006 enhances its own effects [29]. Kinetic studies demonstrated that optimal concentrations of CpG2006 combined with IL-2 provide maximal cell stimulation after 2–5 days (data not shown). These observations are in line with cell-cycle analyses demonstrating that, in the same time frame, more than 95% of B cells enter the cell cycle independently from their memory or naïve phenotype [24,30].

The SEQUENTIAL method does not modify the expression of CD21, which is the receptor for EBV [31].

The next step was to correlate the efficiency of EBV infection to the expression of CD23, which is directly upregulated by EBNA2, one of the earliest markers expressed by EBV-infected B-lymphocytes [32], it is expresses at high levels on EBV-transformed B cells [33] and its expression correlates with IgG secretion [34]. The results demonstrated that CD23 expression (the number of positive cells and mean fluorescence intensity) is higher on cells exposed to the polyclonal activators and EBV separately than on those exposed simultaneously (Figure 2B).

The B-lymphocytes representing the whole repertoire of a selected donor immortalized with the SEQUENTIAL method show a significantly higher viability than those obtained with the COMBINED method, and in consequence are easily clonable by limiting dilution, either in the presence or in the absence of polyclonal activators, such as CpG2006 and IL-2. TLR9 expression is increased after EBV infection. Thus bona fide, the addition of CpG2006 during the cloning phase enhances cell growth. However, we observed that cloning efficiency may be independent of CpG2006 and IL-2 in some donors, suggesting that it is also dependent on the genetic background or physiological state of the donor. This issue warrants further investigation. The presence of CpG2006 and IL-2 during the cloning phase may also have practical relevance for screening procedures. For instance, trace amounts of CpG2006 completely block HCMV infection *in vitro*, hampering screening by functional assays [20]. Moreover, CpG2006 is a potent inducer of cytokines, such as IL-12 and IFN-γ, potentially interfering with a number of bioassays [35].

The validity of the SEQUENTIAL method was evaluated by using it in a project to produce neutralizing human mAbs against HCMV, a leading cause of morbidity and mortality in immunocompromised individuals and the most serious pathogenetic agent in transplant patients [15,36,37]. In the absence of other therapies such as vaccines [38], these patients are treated with antivirals, which are often associated with considerable side effects and the emergence of drug-resistant strains. HCMV is also the leading viral cause of congenital infection associated to significant birth defects and neurological damage for which no effective therapies are available before birth [13,39]. Hyperimmune globulins specific for HCMV are beneficial in both conditions for the prevention and treatment of these infections [40,41]. Further, transfer of memory B cells from immune animals to severely immunodeficient recipients confers long-term protection from lethal murine CMV infection. This indicates that the humoral immune response is effective against CMV infection in the absence of a T cell-mediated response [42]. Natural human mAbs with stronger neutralizing activity than that of currently available immunoglobulin preparations may be a decisive alternative in the prevention of primary and re-activated HCMV infections in immunosuppressed patients, or during high-risk pregnancies [43].

Using the SEQUENTIAL method, we obtained natural antibodies neutralizing HCMV infectivity by immortalizing B-lymphocytes from a healthy donor (CMV5) and from a patient recovering from acute HCMV infection (CMV7). High titers of anti-HCMV-specific IgG coupled with the virus-neutralizing activity of the sera were the criteria for selecting the two donors from a larger panel (Table 1). The immortalized B-lymphocytes were cloned by limiting dilution. After 3 weeks, clusters of cells growing in wells were appreciable and the IgG concentration in the clone supernatants was 10–40 μg/ml/10^6 ^cells, as determined by means of an immunoenzymatic method, measuring IgG concentrations in the media normalized for cell densities. Clones producing HCMV-specific IgG were identified by different primary screening assays. In a first experiment a total of 1,664 clones were tested either by their binding to specific HCMV envelope glycoprotein domains (such as those from gB or gH, which have been shown to induce neutralizing antibodies) [44], or by their ability to neutralize HCMV infectivity *in vitro *[45,46]. The effect on HCMV infectivity was determined by an HCMV microneutralization assay based on the evaluation of the ability of clone supernatants to interfere on the infectivity of HCMV AD169 strain (a laboratory strain from ATCC, cod. VR-538) in human Embryo Lung Fibroblasts (HELF). This strategy yielded 44 clones producing mAbs specific for the gB, and 15 for gH. Nine of these displayed a neutralizing activity of > 40%. Those supernatants that were negative in the gB and gH ELISAs, or in the neutralization assay were also screened in an ELISA that detects human IgG that bind to HCMV virion proteins extract (BEIA-CMV), and 8 samples were further selected by this screening assay. In a second experiment, the immortalized B-lymphocytes were cloned by limiting dilution and after 3 weeks, 324 clones were screened directly for their neutralizing activity. Of these, 20 produced IgG with a neutralizing activity of > 40%, among these, 2 wells were found to bind gB, while the remaining 18 did not bind in either the gB or gH domains.

Four clones has been chosen as promising candidates for future clinical applications on the basis of their intrinsic characteristics: 10B7 and 8C10 showed a very strong binding to gB glycoprotein and 1F7 to gH glycoprotein; 26A1 has the strongest neutralizing activity. All clones were expanded in the absence of CpG2006, IL-2 and a feeder layer, and further characterized. MAbs10B7 and 8C10 proved to be specific for the HCMV envelope glycoprotein gB, mAb 1F7 for the HCMV envelope glycoprotein gH and mAb 26A1 recognized a viral antigen not yet characterized, as inferred from the reactivity on the HCMV virion proteins extract (Table 2).

All of the antibodies neutralized the infectivity of the HCMV AD169 laboratory strain in human HELF as well as that of the VR1814 clinical isolate in human umbilical vein endothelial cells (HUVEC), indicating that they are neither strain nor cell-specific. Lastly, none of the antibodies bound the HELF or HUVEC (data not shown). The specificity of the HCMV neutralizing activity was confirmed by the lack of significant neutralization of herpes simplex virus (HSV)-1 in Vero cells (data not shown).

**Figure 3:**
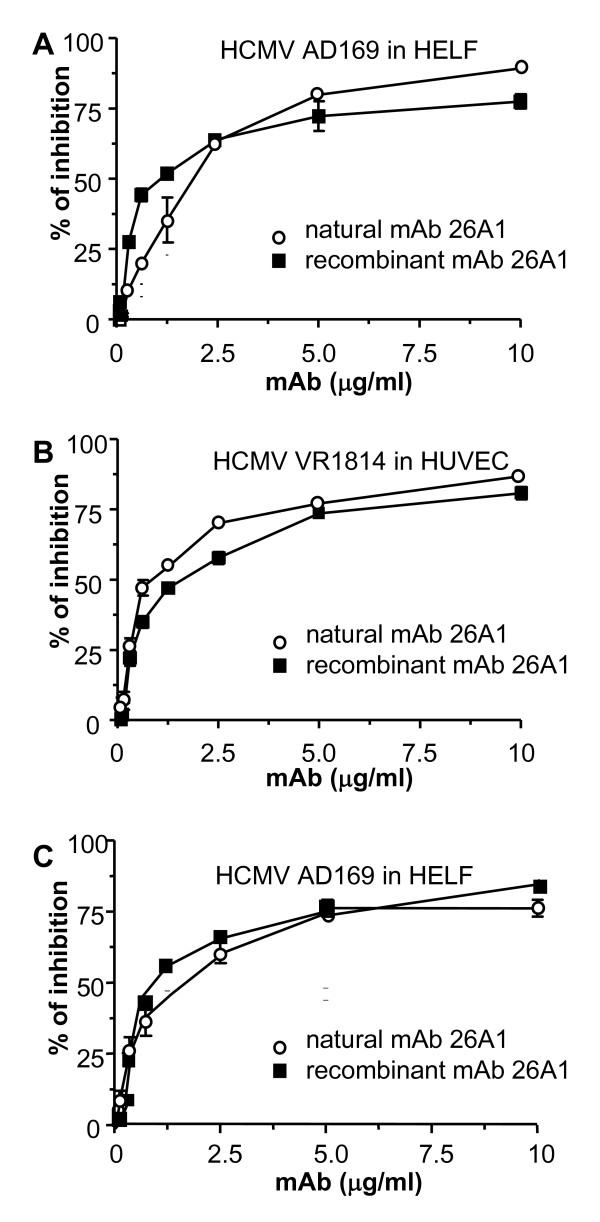
**Neutralizing activity of natural (■) and recombinant (○) purified 26A1 IgG**. The 26A1 mAb was purified from serum-free supernatants by protein A chromatography. The neutralizing activity of HCMV infectivity was then tested either with the laboratory strain AD169 in HELF (panel A) or with the clinical isolate VR1814 in HUVEC (panel B). The effect of 26A1 IgG on HCMV infectivity was measured by staining the HCMV IEA by indirect immunoperoxidase. Five fields (HPF 20×) were counted per well from triplicate wells. Percentage of inhibition of viral infectivity was calculated using the following formula: [(means ± s.d. of IEA^+ ^nuclei of HCMV infected cells – means ± s.d. of IEA^+ ^nuclei of 26A1-treated HCMV infected cells/means ± s.d. of IEA^+ ^nuclei of HCMV infected cells) ×100]. Results represent the means ± s.d. of 3 independent experiments. Plaque reduction assay (panel C) shows the neutralizing activity of natural and recombinant 26A1 mAb against HCMV AD169 (1,000 pfu/rxn) in HELF. The values were plotted as a function of IgG concentration, and the concentrations of IgG required to inhibit viral infection by 50% (IC_50_) were calculated by linear regression using GraphPad Prism (v. 4.0) software.

Clone stability was tested by maintaining the cells for 3 months in serum-free medium without CpG2006 or other additives, and the mAbs recovered in the supernatant at a concentration of 20–50 μg/ml. All purified IgG maintained their neutralizing activity; mAb 26A1 demonstrated a potent neutralizing capacity, with a calculated inhibitory concentration 50 (IC_50_) of 0.92 μg/ml against AD169 in HELF (Fig. 3A), and of 1 μg/ml against VR1814 in HUVEC (Fig. 3B). The potency of 26A1 against both HCMV strains was ~20 fold higher than the reference anti-HCMV IgG (IC_50 _of 16 μg/ml), currently used in transplant recipients. The inhibitory activity of mAb 26A1 against the whole viral replicative cycle was confirmed by plaque reduction neutralization assay (Figure 3C). The mechanism of virus neutralization is subject of ongoing studies.

**Table 1:** Characterization of HCMV Donor Sera

**Donor**	**BEIA-CMV^a^**	**gB^b^**	**CMV neutralization titer^c^**
**CMV5**	+	+	1:42
**CMV6**	+	-	1:12
**CMV7**	+	+	1:105
**CMV8**	+	-	1:20
**CMV9**	-	-	1:15

a. BEIA CMV ELISA detects human IgG that bind to total protein lysates from HCMV virions.

b. gB ELISA detects human IgG that bind to the AD-2 region of the HCMV envelope glycoprotein gB.

c. Sera were tested in the HCMV microneutralization assay using the AD169 strain and HELF [48]. Numbers indicate dilution of serum required for 50% inhibition of HCMV infectivity.

**Table 2:** Characterization of clones producing neutralizing IgG to HCMV

	**10B7**	**1F7**	**26A1**	**8C10**
**Donor**	CMV5	CMV5	CMV7	CMV7
**Doubling Time (days)**	4	4	4	4
**IgG Subclass**	IgG1	IgG1	IgG1	IgG2
**BEIA-CMV^a^**	-	-	+	+
**gB ELISA**	+	-	-	+
**gH ELISA**	-	+	-	-
**HELF binding^b^**	-	-	-	-
**HUVEC binding^b^**	-	-	-	-
**HCMV neutralization^c^**	64.72 ± 11.34	47.23 ± 2.98	86.34 ± 8.15	52.3 ± 13.34

a. Antibodies were tested using the BEIA CMV ELISA assay

b. Antibodies were tested for binding to uninfected HELF and HUVEC by immunohistochemistry.

c. Antibodies were tested using the HCMV microneutralization assay, with the HCMV AD169 strain and HELF. Results are expressed as % of inhibition of virus infectivity. Data represent the mean ± s.d. of 3–4 experiments.

**Figure 4:**
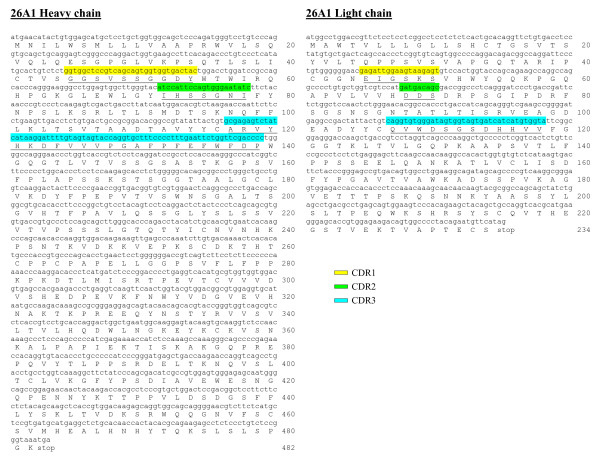
**Alignment of the DNA (lower case) and protein (upper case) consensus sequence of the heavy and light chains of the human recombinant 26A1 IgG**. The variable regions of heavy chain (VH) and light chain (VL) of mAb 26A1 were sequenced according the technology established by Fusion Antibodies Ltd. and described in the Methods section.

The broad and robust neutralizing activity of mAb 26A1 encouraged us to generate recombinant IgG suitable as prophylactic or therapeutic tools in clinical applications. To this aim, cells were submitted for 5' RACE PCR amplification, the PCR products of two amplification reactions were cloned using a *Eco RI *restriction site in a sequencing vector and used for transforming TOP10 *E. coli*. A single VH and VL sequence was obtained from each cell culture, confirming that each original culture was clonal (Figure 4).

The sequences encoding the VH and VL regions of mAb 26A1 were cloned in expression vectors for the expression of mAb 26A1 variable regions as recombinant IgG1. The recombinant mAb 26A1 confirmed the neutralizing activity of the natural mAb 26A1 (Figure 3).

The SEQUENTIAL method is also effective when used with frozen cells. Indeed, it was possible to select antibodies neutralizing HSV-1 and HSV-2 infectivity from frozen PBMCs from an HSV and HIV co-infected individual (not shown).

## Conclusion

In summary, this study provides proof of concept that good-quality, fully human mAbs with desired specificity and biological activity can be generated with the SEQUENTIAL method. The strengths of this approach are: i) it allows the selection of human monoclonal IgG to a variety of antigens, from a small sample of fresh or frozen peripheral blood, ii) it is rapid, iii) screening can be performed using a variety of assays, including functional assays, iv) the mAbs of interest can be easily produced from the original clone as recombinant proteins suitable for clinical applications, and v) the generation of IgG-secreting polyclonal populations can be considered as a library of antibody-secreting cells that can be used to select mAbs with specificities not considered when cells were immortalized.

The technology described has proven to be more reproducible, efficient and rapid than previously reported techniques, and can be adopted at low overall costs by any cell biology laboratory for the development of fully human mAbs for immunotherapeutic uses. It can be applied to the isolation of natural mAbs from individuals affected by a variety of diseases, ranging from infectious diseases to cancer and autoimmune diseases. It allows the isolation of a large repertoire of human mAbs with desired specificities to be selected from a small (~8 ml) volume of peripheral blood, and can be used with either fresh or frozen B-lymphocytes. Moreover, direct cloning by limiting dilution and robust IgG production allow the generation of panels of mAbs with different specificities, thus allowing the development of mAb cocktails for immunotherapy [47].

## Methods

### Collection of Blood Samples and Isolation of Mononuclear Cells

After informed consent was obtained, peripheral blood (8–10 ml) was collected from healthy blood donors or infectious disease patients. Serum was screened for reactivity with HCMV glycoproteins and for HCMV neutralizing activity. Peripheral blood mononuclear cells (PBMCs) were purified from heparinized peripheral blood by density gradient centrifugation on Ficoll/Hypaque (Amersham Pharmacia, Milan, Italy).

### Qualitative and quantitative comparison of different EBV transformation methods

CD22^+ ^lymphocytes were purified by magnetic selection (Miltenyi Biotec, Bologna, Italy) from PBMCs pooled from 5 healthy donors, then CD22^+^cells were divided into three aliquots that were exposed to different EBV-based methods for immortalization. For the BASIC process, CD22^+^/IgG^+ ^lymphocytes were isolated by depletion of IgM^+ ^cells with a MoFlo^® ^High-Performance Cell Sorter (Dakocytomation, Glostrup, Denmark) and cultured (10^6^/ml) in 24 well plates in Iscove's Modified Dulbecco's medium (IMDM) supplemented with L-glutamine, 100 μg/ml streptomycin and 100 U/ml penicillin, non-essential amino acids and 10% FBS (complete medium) for 15 h, in the presence of EBV-containing supernatant (from B95-8 marmoset lymphoma cells, ATCC, Washington, DC, cat. no. CRL-1612) 1:1 (v/v) at 37°C and 5% CO_2_. The cells were then washed and cultured (1.5 × 10^6^/ml) for 10 days in complete medium in the presence of irradiated (30 Gy) allogeneic PBMCs (0.5 × 10^6^/well). For the COMBINED process, CD22^+^/IgG^+ ^lymphocytes (selected as above mentioned) were cultured (1.5 × 10^6^/ml) in complete medium with EBV-containing supernatant 1:1 (v/v), CpG ODN2006 (5'-TCGTCGTTTTGTCGTTTTGTCGTT-3') (1 μg/ml) (Metabion, Planegg-Martinstried, Germany) and IL-2 (200 U/ml) (Roche, Milano, Italy), in the presence of irradiated, allogeneic PBMCs. For the SEQUENTIAL process, CD22^+ ^lymphocytes were cultured (1.5 × 10^6^/ml) for 4 days in complete medium containing CpG2006 (1 μg/ml) and IL-2 (200 U/ml), then washed. The IgG^+ ^lymphocytes were isolated by depletion of IgM^+ ^cells with a MoFlo^® ^High-Performance Cell Sorter (Dakocytomation, Glostrup, Denmark), cultured (10^6^/ml) in complete medium with EBV-containing supernatant 1:1 (v/v) for 15 h, washed and maintained (1.5 × 10^6^/ml) in complete medium in the presence of irradiated, allogeneic PBMCs.

The number of viable lymphoblasts was measured microscopically by trypan blue dye exclusion. Where indicated, the DNA intercalating, fluorescent dye propidium iodide (PI, Sigma Aldritch) was used with a FACSCalibur flow cytometer and CellQuest Software (Becton Dickinson, Franklin Lakes, NJ) to assess cell viability. Viable cells were defined as those with a high forward and orthogonal scatter, characteristic of lymphoblasts, and excluding PI.

### Analysis of CD23 expression

CD23 expression was measured by direct immunofluorescence. Briefly, cells (3 × 10^5^/100 μl) were incubated for 30 min at 4°C with anti-human CD23-FITC (Becton Dickinson, Milano, Italy). After washing, fluorescence was analyzed using a FACSCalibur flow cytometer and CellQuest software. CD23 staining was evaluated only on the viable lymphoblast population, gated using forward/orthagonal scatter and PI staining, as described above. Background staining was determined with a FITC-conjugated isotype-matched negative control mAb. The number of cells analyzed was 10,000.

### HCMV microneutralization assay

The microneutralization assay was adapted from a previously reported technique [48]. Briefly, HELF were plated (2.0–2.5 × 10^4^/well) onto flat-bottom wells of a 96-well plate in 100 μl of Minimal Essential Medium (MEM) (Gibco-BRL, Milano, Italy) with 10% FBS, 100 U/ml penicillin and 100 μg/ml streptomycin (growth medium) and cultured for 24 h at 37°C. Fifty μl of supernatant from B cell clones (or purified IgG at indicated concentration) were incubated with the laboratory strain HCMV [AD169; 500 plaque forming units (pfu) in 50 μl of MEM with 5% FBS] for 1 h at 37°C, then added to the fibroblast monolayers. The plates were then centrifuged at 2,000 g for 30 min and incubated for 90 min at 37°C in 5% CO_2_. Growth medium (100 μl) was added and the cultures incubated for 72 h.

The effect of B cell supernatants (or purified IgG) on HCMV infectivity was measured by staining the HCMV Immediate Early Antigens (IEA, IE1+IE2) by indirect immunoperoxidase. IEA-positive nuclei were counted under the microscope. B cell supernatants containing irrelevant IgG antibodies were used as a negative control and a commercial preparation of human IgG, purified from the serum of HCMV seropositive patients (Cytotect; Biotest, Dreieich, Germany), as a positive control, respectively. Positivity was defined as ≥ 40% inhibition of IEA^+ ^cells, compared to negative control wells. HCMV microneutralization assays were also performed with the endotheliotropic HCMV VR1814, a derivative of a clinical isolate recovered from a cervical swab of a pregnant woman [49], and HUVEC, as described above. Experiments were performed with cells at passage 2–6. Plaque reduction assay was performed as reported [50].

Selected experiments were done with IgG purified on protein A columns (Biorad, Milano, Italia) from serum-free supernatants (natural IgG).

### ELISA assay for detection of IgG binding to the total HCMV virion proteins and to the gB and gH envelope glycoproteins (gB ELISA and gH ELISA)

Antibodies were tested using the ELISA assay for detection of IgG binding to total proteins extract from HCMV virions (BEIA CMV IgG Quant Kit; Bouty, Milano, Italy) according to the manufacturer's instructions. The anti-HCMV recombinant gB IgG ELISA was purchased from Biotest, AG (Dreieich, Germany) and used according to the manufacturer's instructions. The ELISA makes use of the recombinant autologous interstrain fusion antigen CG3, corresponding to a combination of the gB antigenic domain 2 (AD2) from HCMV strains AD169 (SwissProt Acc. No. P06473) and Towne (SwissProt Acc. No. P13201). The AD2 region contains a site (amino acids 70–81) conserved in different viral strains, that has been shown to be recognized by neutralizing antibodies [51,52]. The gB-GST antigen was purchased from Biodesign (Saco, ME, USA) and contains a gB immunodominant region fused to GST that reacts with HCMV-positive sera. It was used in ELISA assays according to the manufacturer's instructions.

The anti-HCMV recombinant gH-specific ELISA was performed using the recombinant gH-GST antigen coated onto plastic. gH-GST corresponds to an in-frame fusion between the gH amino terminal region (amino acids 16–144) from the HCMV strain VR1814 and GST. The amino terminus of gH contains a linear antibody binding site between residues 34–43 that is recognized by neutralizing antibodies [44,53]. The gH gene segment was PCR amplified from VR1814 DNA with the following primers: VR1814 gH F: 5'-AGTCATGGATCCCTCCTTAGTCACCTCA-3'; VR1814 gH R: 5'-ACTGATCTCGAGGCCTTCAGCTGCTGC-3'. The 400 bp PCR product was then digested by *XhoI *and *BamHI*, the restricted fragment purified by agarose gel elecrophoresis and cloned into the pGEX 4T3 expression vector (Amersham) that had been restricted by *XhoI *and *BamHI *and gel purified. The recombinant gH-GST fusion protein was then expressed in *E. coli *BL21 and purified from the bacterial cell lysate on the basis of GST affinity.

### Sequence of 26A1 IgG and Expression of 26A1 VH/VL Sequences

The variable regions of heavy chain (VH) and light chain (VL) of mAb 26A1 were sequenced according the technology established by Fusion Antibodies Ltd. Briefly, frozen cells pellets (~5 × 10^4 ^cells) were used for extracting total RNA with STAT-60 RNA extraction reagent. cDNA was produced by reverse transcription with an oligo(d)T primer. PCR reactions were set up to amplify VH and VL regions with a mix of IgG- and Igk/λ specific primers, respectively. The PCR products of two amplification reactions were cloned using a *Eco RI *restriction site in a sequencing vector (pCR2.1; Invitrogen) and used for transforming TOP10 *E. coli *cells, following the manufacturer's instructions. A consensus sequence was determined from at least ten clones. The resulting DNA sequences were aligned and translated into protein sequences enabling the generation of consensus DNA and protein sequence for VH 26A1 and VL 26A1. The VH 26A1 and VL 26A1 protein sequences were compared and aligned with sequences present in the GenomeQuest, GeneSeq, and EBI databases. The CDRs characterizing VH 26A1 and VL 26A1 protein sequences were predicted by the IMGT database [54].

To generate a recombinant IgG1, a consensus 26A1 VH sequence was amplified by PCR with the following primers: 5'-TTTTTTAGATCTCACCATGAACATACTGTGGAGCATGCTC-3'; 5'-TTTTTTAGATCTTGAGGAGACGGTGACCAGGGTTCC-3'. The primers contained a BglII restriction site (underlined) for ligation into the hIgG expression vector already containing human IgG1 heavy chain constant domains. The in-house designed bicistronic mammalian retroviral expression vector pRetMEx (Fusion Antibodies Ltd. Belfast, Ireland) was a dual promoter vector allowing expression of both antibody chains. The VH domain was cloned into the BamH1 site of multiple cloning site (MCS) 1 of the vector.

A complete 26A1 recombinant vector was confirmed by DNA sequencing. CHO DG44 cells were adapted to serum-free suspension culture and seeded at 1 × 10^6 ^cells/ml in 125 ml spinner flasks. 300 μg of plasmid DNA was mixed with 900 μg of linear 25 kDa PEI and used to transfect the cell culture. The medium was harvested after 6 days.

Recombinant mAb was purified with a Protein G column on an Akta Prime chromatography unit following the manufacturer's standard programme (recombinant IgG).

### Statistical analysis

Values are expressed as means ± sd. Data were analyzed for significance using a one-way analysis of variance (ANOVA) with Bonferroni post-test correction for multiple comparisons. Data were considered significant at P < 0.05.  

## Competing interests

A. Funaro, M. Murphy and G. Garotta are inventors on a PCT patent application (published as WO 2007/068758) describing the technology. A Funaro, G. Gribaudo and S. Landolfo are inventors on unpublished patent applications describing specific HCMV-neutralizing antibodies that were isolated using the described technology. All patent applications are filed in the name of Ribovax Biotechnologies SA (Petit-Lancy, Switzerland).

Data were analyzed for significance using a one-way analysis of variance (ANOVA) with Bonferroni post-test correction for multiple comparisons. Data were considered significant at *P *< 0.05.

## Authors' contributions

AF, GGr, MM and GGa designed the study. AL carried out the neutralization assays, NLB carried out the immunoassays, EO participated to the analysis and interpretation of data. EV, LC. selected antibodies neutralizing HSV-1 and HSV-2, SL and LF contributed to design the study. RB prepared the recombinant 26A1 IgG, and AF and FM wrote the paper. All authors read and approved the final manuscript.
